# Idiopathic Submitral Left Ventricular Aneurysm: an unusual substrate for ventricular tachycardia in Caucasians

**Published:** 2005-07-01

**Authors:** Babak Kazemi, Arash Arya, Majid Haghjoo, Mohammad Ali, Mohammad Ali Sadr-Ameli

**Affiliations:** Department of Pacemaker and Electrophysiology, Rajaie Cardiovascular Medical Center, Iran University of Medical Sciences, Mellat Park, Vali-e-Asr Avenue, Tehran, 1996911151 Iran

**Keywords:** submitral aneurysm, ventricular tachycardia

## Abstract

Annular submitral aneurysms have been rarely reported in Caucasians. They are typically diagnosed in non-white adults who present with severe mitral regurgitation, heart failure, systemic embolism, ventricular arrhythmias, and sudden cardiac death. In this article, we describe the case of a white woman, presenting with ventricular tachycardia, who had a large submitral left ventricular aneurysm diagnosed incidentally during coronary angiography.

## Introduction

In 1962, Abrahams et al. described 12 patients, with an unusual form of left ventricular aneurysm, which they termed “annular subvalvular left ventricular aneurysm“ [1]. These aneurysms are of two types: subaortic and submitral. Submitral aneurysm (SMA) is a congenital outpouching of left ventricular (LV) wall, invariably occurring adjacent to the posterior leaflet of mitral valve [2]. SMA is typically diagnosed in young adults who present with severe mitral regurgitation, heart failure, systemic embolism, ventricular arrhythmias, and sudden cardiac death. African blacks account for the majority of the SMA cases reported in world literature, with an estimated incidence of 34 per 10,000 cardiovascular illnesses. About 15 cases of SMA have been reported from India [3]. Few cases of SMA have been reported in Caucasians, all accompanied by significant mitral regurgitation [4,5,6].

Our patient is a rare case of a large SMA without involvement of the mitral valve apparatus, in a female who presented with sustained monomorphic ventricular tachycardia (SMMVT).

## Case Report

**Figure 1:**
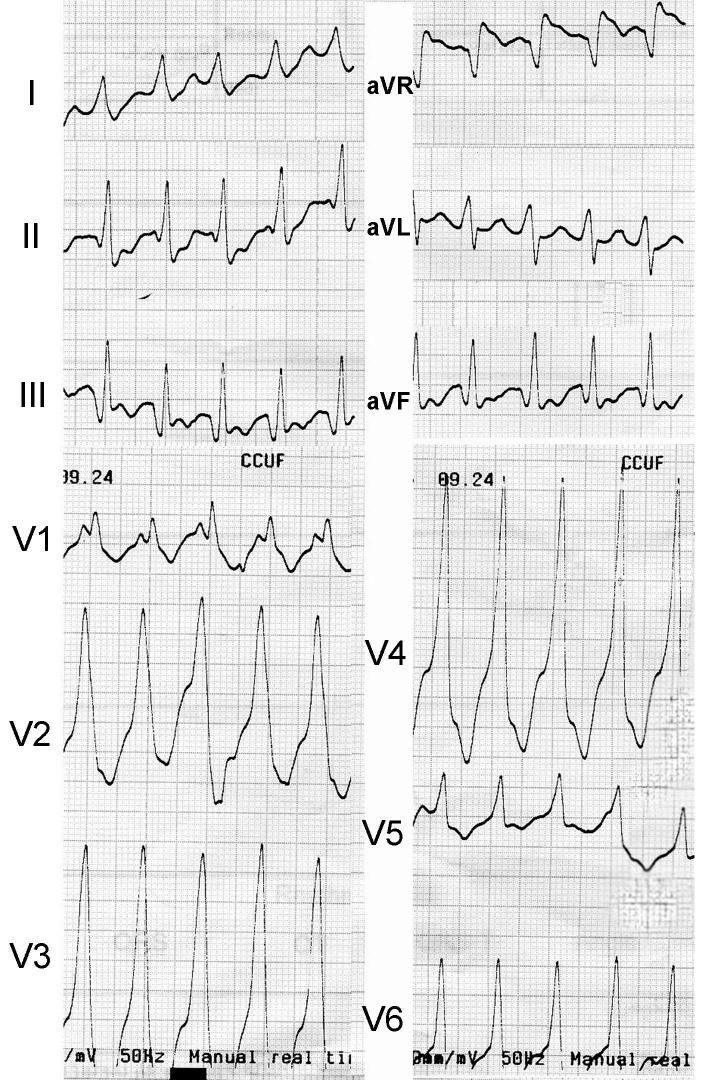
Twelve-lead surface ECG during ventricular tachycardia with a right bundle branch blocks pattern

**Figure 2:**
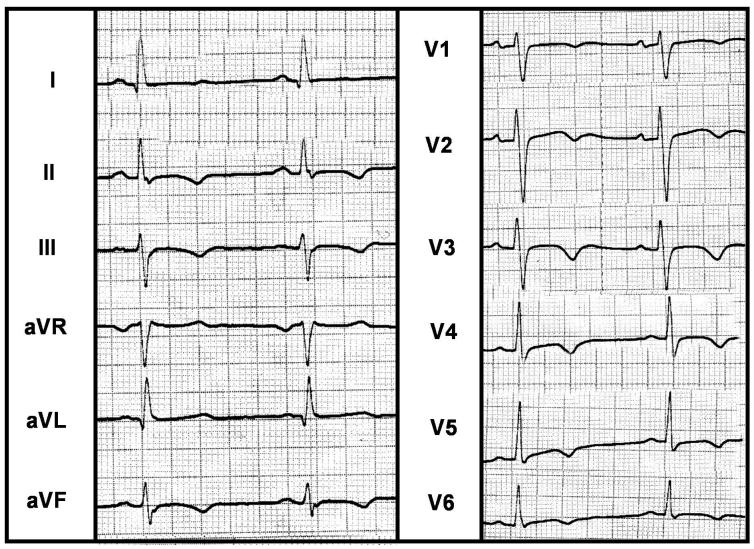
Twelve-lead surface ECG during sinus rhythm. Note low heart rate, long QT interval, and diffuse T wave changes

A 63-year-old female presented to our institution for recent onset of paroxysmal palpitation and presyncope. At the emergency department initial evaluation revealed a rapid pulse rate and the 12-lead electrocardiogram (ECG) showed a wide QRS complex tachycardia, with a right bundle branch block morphology, compatible with VT (Figure 1). This arrhythmia was successfully converted by 200 J external cardioversion. She was diabetic, with a long history of hypertension and mild hyperlipidemia. Her medications included atenolol 100 mg/d, amiodarone 200 mg/d, enalapril 5 mg/bid, and glibenclamide 5 mg/bid. Physical examination was unremarkable. The 12-lead ECG during sinus rhythm showed sinus bradycardia, with a long QT interval (QTc=568 msec, probably because of amiodarone; QTc before starting amiodaron was normal) and diffuse nonspecific T wave inversion in inferior and all the precordial leads (Figure 2). Chest radiogram (CXR) revealed no abnormality. In echocardiography, LV was mildly dilated (LVEDD=60mm), with mild dysfunction (LVEF=45%), mild isolated septal hypertrophy and posterior wall thinning (IVS=14.4mm, PW=6mm). There was no significant valvular lesion, and right heart chambers had normal dimensions and function. In laboratory evaluation, blood sugar and creatinine were mildly elevated, there was no electrolyte or acid-base abnormality, and cardiac specific enzymes were normal.

Her past medical history was remarkable for her presentation with atypical chest pain, palpitation, and presyncope, nine years ago. Myocardial perfusion imaging at that time, revealed a perfusion defect in the anteroseptal wall, and 24 hr-Holter monitoring showed episodes of nonsustained ventricular tachycardia. Coronary angiogram showed normal epicardial coronary arteries. LV angiography was omitted because of relatively good LV function and no evidence of valvular heart disease at echocardiographic study. Tilt-test was done for a history of presyncope. At the active stage, after receiving nitroglycerin spray, she developed a hemodynamically stable sustained monomorphic ventricular tachycardia, which was controlled with intravenous lidocaine. She was recommended oral amiodaron after discharge from hospital, and had been asymptomatic until several days before her recent presentation.

**Figure 3:**
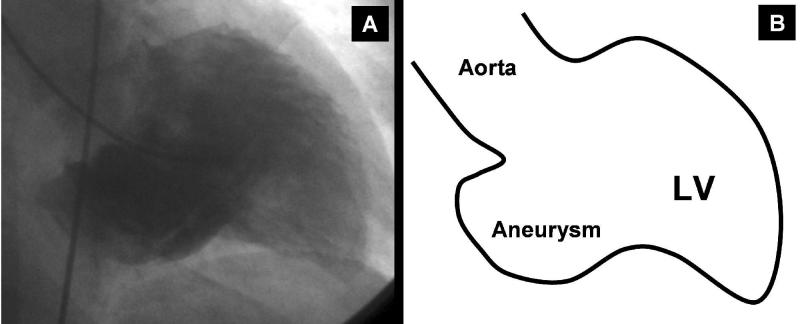
Ventricular angiogram in the right anterior oblique projection, showing the large posterobasal aneurysm and mild Left ventricular enlargement. **A**: the fluoroscopic view; **B**: the schematic view.

According to her past history and the presenting symptoms, coronary angiography with left heart catheterization was repeated, which revealed patent epicardial arteries, with a large posterobasal aneurysm, mild LV enlargement, no  mitral regurgitation, and a global LVEF of about 45% (Figure 3).

## Discussion

LV aneurysm in the absence of coronary artery disease is rare [7], but they may occur in congenital, traumatic, connective tissue, primary myocardial, or infective heart disease. Chagas’ disease, infective endocarditis,  and idiopathic or viral myocarditis  have caused LV aneurysms of infective origin [3,8,9].

Among LV aneurysms, the subvalvular types are the least common [8]. Because of the anatomy of the mitral annulus, congenital aneurysms occur only below the posterior leaflet. Two-thirds of the mitral annulus is related to the posterior leaflet which is attached to the myocardium of LV by annular tissue (mitral ring). The immediate external relationship of the mitral ring is the epicardium in the atrioventricular groove. A dehiscence of this muscular-fibrous union will result in a SMA below the posterior leaflet [8]. Morphologically identical aneurysms may occur in the same position following surgical replacement of the mitral valve [8].   The remaining one-third of the mitral annulus is formed by the fibrous union between the aortic and mitral valves through the so called “mitral-aortic intervalvular fibrosa”. Perforation of the latter structure has been described only as a consequence of infective endocarditis [8].   The exact nature or cause of the defect in subvalvular aneurysms is uncertain. Virtually all the cases have been described in black people, few in other ethnic groups, but least in Caucasians [6]. The predilection for constant anatomic sites in the absence of evidence of coronary atherosclerosis, infection, or trauma would favor a congenital cause. Subvalvular aneurysms have not hitherto been reported with another congenital cardiac anomaly. Although their pathogenesis appears to be similar, it is uncertain, however, whether the dehiscence between the annulus and the related structure is a primary failure of union or a later spontaneous separation.

Patients with SMA usually present with sudden, acute, left-sided heart failure, with an insidious appearance of cardiac decompensation or with angina. Most frequently, the lesion is detected incidentally in an otherwise asymptomatic person. Associated valvular regurgitation is almost invariably present in these patients [4]. Ventricular tachycardia as the presenting manifestation has also been reported in patients with SMA [5,6,10,11,16]. In a recent study, the majority of idiopathic LV aneurysms with SMMVT were restricted to the posterior and/or inferior wall [12].

The diagnosis of this condition presents few difficulties in cases with anterolateral aneurysms, in which the combination of an aneurysmal pulsation in the third intercostal space with a mitral systolic murmur, a chest radiograph showing an obvious aneurysmal bulge, and an abnormal ECG in a young black patient is quite typical. Difficulties, as in our case, do arise when the aneurysm has a posterior origin and does not produce abnormal pulsation or an aneurysmal bulge in the postero-anterior chest radiograph.

Considering the nonspecific, diffuse negative T waves with QT prolongation and the mild LV dysfunction in our patient, we could not exclude the possibility of transient myocardial ischemia, due to coronary vasospasm or recanalized coronary thromboembolism [13]. Although, the former seems unlikely in the absence of cardiac enzyme elevation, no pathologic Q wave in the ECG, and relatively preserved LV function. On the other hand, an inflammatory origin also seems unlikely, considering the patients past history, the morphologic characteristics of inflammatory aneurysms (small size, sometimes multiple), and rarity of preserved LV function in this condition (3.3%) [9]. The lack of systemic and constitutional manifestations, no history of trauma, and the normal CXR places other diagnosis at the most remote possibilities.

An accurate diagnosis can be made by left ventricular angiography [4].  Although the aneurysm can also be detected noninvasively by echocardiography, even in expert hands, it can be missed, especially if it is located in the posterior wall [4,9]. Magnetic resonance imaging is a promising tool for diagnosing these aneurysms [14].

SMAs may rupture spontaneously [15] or be the origin of systemic emboli [4], and malignant ventricular arrhythmias [5,6,10,11,16]. The definite treatment is surgical [3], but in high surgical risk patients, medical treatment, and implantable cardioverter-defibrillators in cases with malignant ventricular arrhythmias, should be considered [16].

Our patient had clinical characteristics relatively similar to the previously reported cases, but the occurrence of SMA in a Caucasian woman, with no mitral valve insufficiency or signs of heart failure and presenting with a malignant ventricular arrhythmia, is quite unique.
